# Identification of CD160-TM as a tumor target on triple negative breast cancers: possible therapeutic applications

**DOI:** 10.1186/s13058-024-01785-x

**Published:** 2024-02-15

**Authors:** Claire Scheffges, Jérôme Devy, Jérôme Giustiniani, Stessy Francois, Lucille Cartier, Yacine Merrouche, Arnaud Foussat, Stéphane Potteaux, Armand Bensussan, Anne Marie-Cardine

**Affiliations:** 1 INSERM U976, HIPI, Team 1, 75010 Paris, France; 2 Université Paris Cité, IRSL, 75010 Paris, France; 3 Alderaan Biotechnology, 75005 Paris, France; 4 UMR CNRS/URCA 7369, MEDyC, Université de Reims-Champagne-Ardennes, 51100 Reims, France; 5 INSERM U955, 94000 Créteil, France; 6 Département de Recherche, Institut Godinot, 51100 Reims, France; 7 UR7509, IRMAIC, Université de Reims-Champagne-Ardennes, 51097 Reims, France

**Keywords:** TNBC, CD160-TM, Tumor antigen, Antibody-based therapy

## Abstract

**Background:**

Despite major therapeutic advances, triple-negative breast cancer (TNBC) still presents a worth prognosis than hormone receptors-positive breast cancers. One major issue relies in the molecular and mutational heterogeneity of TNBC subtypes that is reinforced by the absence of reliable tumor-antigen that could serve as a specific target to further promote efficient tumor cell recognition and depletion. CD160 is a receptor mainly expressed by NK lymphocytes and presenting two isoforms, namely the GPI-anchored form (CD160-GPI) and the transmembrane isoform (CD160-TM). While CD160-GPI is constitutively expressed on resting cells and involved in the generation of NK cells' cytotoxic activity, CD160-TM is neo-synthesized upon activation and promotes the amplification of NK cells' killing ability.

**Methods:**

CD160 expression was assessed by immunohistochemistry (IHC) and flow cytometry on TNBC patient biopsies or cell lines, respectively. Antibody (Ab)-mediated tumor depletion was tested in vitro by performing antibody-dependent cell cytotoxicity (ADCC) and phagocytosis (ADCP) assays, and in vivo on a TNBC mouse model.

**Results:**

Preliminary data obtained by IHC on TNBC patients' tumor biopsies revealed an unconventional expression of CD160 by TNBC tumor cells. By using a specific but conformation-dependent anti-CD160-TM Ab, we established that CD160-TM, but not CD160-GPI, was expressed by TNBC tumor cells. A conformation-independent anti-CD160-TM mAb (22B12; muIgG2a isotype) was generated and selected according to pre-defined specificity and functional criterions. In vitro functional assays demonstrated that ADCC and ADCP could be induced in the presence of 22B12, resulting in TNBC cell line apoptosis. The ability of 22B12 to exert an in vivo anti-tumor activity was also demonstrated on a TNBC murine model.

**Conclusions:**

Our data identify CD160-TM as a tumor marker for TNBC and provide a rational for the use of anti-CD160-TM antibodies as therapeutic tools in this tumor context.

**Supplementary Information:**

The online version contains supplementary material available at 10.1186/s13058-024-01785-x.

## Introduction

Breast cancer (BC) is the most commonly diagnosed and the first leading cause of cancer death for women worldwide [1]. It is a heterogeneous disease that can be classified according to histopathological criterions, staging, genetic alterations or molecular status [2, 3]. Molecular classification based on immuno-histological staining is routinely used and relies on the detection of hormone receptors (HR) expression, specifically estrogen receptor (ER) and progesterone receptor (PR) but also human epidermal growth factor receptor-2 (HER2), or Ki-67 amplification. According to these characteristics, BC are classified in four main subtypes, namely luminal A (ER^+^, PR^+^, HER2^−^, Ki-67^−^), luminal B (ER^+^, PR^±^, HER2^±^, Ki-67 ^±^), HER2^+^ (ER^−^, PR^−^, HER2^+^), and triple negative (ER^−^, PR^−^, HER2^−^) [4].

Triple negative breast cancers (TNBC) account for 15 to 20% of all BC and are characterized by their aggressiveness, invasiveness and proliferative abilities, resulting in a 5-year overall survival of 77% (against 93% for other subtypes). They are also associated to an earlier age of onset than other subtypes and a higher risk of relapse and metastasis [5–8]. Although new therapeutic options appeared over the last years, TNBC patients still face a worse prognosis compared to HR- or HER2-positive subtypes. Conventional cytotoxic chemotherapy remains the standard of neoadjuvant or adjuvant treatment for early stage TNBC [9]. Over the last years, compelling evidences have emerged regarding the inclusion of immune checkpoint inhibitors (ICI) in the regimen of treatments for TNBC, with a strong focus on PD-1/PD-L1 axis [10]. Numerous clinical trials were conducted for assessing the efficiency of anti-PD-1 (pembrolizumab) or -PD-L1 (durvalumab, atezolizumab) antibodies. While PD-1/PD-L1 inhibitors showed marginal efficiency in monotherapy, combining ICIs with adjuvant chemotherapy gave convincing results for metastatic PD-L1^+^ TNBC (e.g. atezolizumab + nab-paclitaxel) [11–14]. Similarly, phase I–III clinical trials investigating the association of durvalumab, atezolizumab or pembrolizumab with various chemotherapeutic agents highlighted the use of pembrolizumab as neoadjuvant therapy for high-risk early-stage TNBC [15–17]. To take into account the mutational and molecular heterogeneity of TNBC, research evaluating the combination of anti-PD-1/PD-L1 antibodies with targeted therapies (e.g. PARP, Akt, VEGFR or CDK4/6 inhibitors) is currently underway and showed promising therapeutic potential [18]. Despite the improvement brought by immunotherapeutic treatments for TNBC, several challenging issues remained to be addressed, such as the mechanisms leading to primary or secondary immune escape, the optimization of a biomarker panel for maximizing individual benefit, as well as the treatments' regimen complexity. In this context, the identification of a specific TNBC tumor marker could bring new or complementary therapeutic options.

CD160 was originally described on cytotoxic NK and CD8^+^ T lymphocytes as a constitutively expressed glycosyl-phosphatidyl-inositol (GPI)-anchored receptor which extracellular domain was mainly composed of an immunoglobulin (Ig)-like domain [19, 20]. Later on, its expression by CD4^+^ T cells, macrophages or activated endothelial cells was reported [21–23]. Its currently identified ligands are non-classical and classical MHC class I molecules and herpes virus entry mediator (HVEM) [24–26]. On NK cells, CD160-GPI has been identified as an activating receptor mandatory for the initiation of a cytotoxic activity towards target cells. Its engagement induces NK cytotoxicity mainly through IFN-ɣ, TNF-α, and IL-6 cytokine release as a result of phosphatidylinositol-3 kinase signaling pathway activation [27–30]. Previous studies regarding the role of CD160-GPI in T cells revealed its functional versatility. Thus, the interaction between HVEM and CD160-GPI can either attenuate the activity of specific CD4^+^ T cell subsets or enhance/down-modulate the activity of CD8^+^ T lymphocytes depending on the pathological immune context [25, 31–33]. Consequently, CD160-GPI is now considered as a potential immune checkpoint for immunotherapy. Accordingly, its relevance as a prognosis marker, but also as a predictive marker of patients' response to immunotherapies, has been recently documented in the context of lung adenocarcinoma, hepatocellular carcinoma, prostate cancer or chronic lymphocytic leukemia [34–39].

Beside CD160-GPI, a transmembrane form (CD160-TM), issued from *CD160* gene alternative splicing, was described as an additional activating receptor that is neo-synthesized upon NK cell activation. Notably its engagement leads to an amplification of NK cell killing activity [40]. Although both isoforms share an identical extracellular domain, CD160-TM was found as able to interact with MHC class I molecules but not HVEM [41]. So far, CD160-TM functions in normal or pathological conditions remained poorly studied, mostly reflecting the lack of tools allowing discrimination between CD160-GPI and CD160-TM engagement.

By screening a panel of solid tumors to evaluate NK cell infiltration by immunochemistry, we unexpectedly observed a strong expression of CD160 in TNBC tumor biopsies. Thanks to the generation of Abs specific for CD160-TM, we identified the TM isoform as a TNBC tumor marker and explore in vivo the potential of CD160-TM targeting for TNBC depletion.

## Material and methods

### Patients and cells

Archival paraffin-embedded tissues were obtained from TNBC patients. All patients provided informed oral consent and a signed non-opposition form. This study was approved by the local ethics committee (Biological Resource Center of Godinot Institute, agreement no 170,551/1371F).

All but one (IJG1731) TNBC cell lines were purchased from the European Collection of Authenticated Cell Cultures/ECACC or the German Collection of Microorganisms and Cell Cultures/DSMZ (MDA-MB-231/HTB-26, MDA-MB-453/HTB-131, BT20/HTB-19; full data sheets available online). IJG1731 cell line was generated in house and identified as a TNBC cell line by flow cytometry (ER^−^PR^−^HER2^−^EGFR^+^ phenotype). In addition, RNA sequencing revealed activating mutations in BRAF (G464V), KRAS (G13D) and TP53 (R280K) transcripts and a moderated but significant amplification of NRAS and TP53 transcripts (3.3 and 3.4-fold, respectively). TNBC cell lines were cultured in RPMI1640 (IJG1731), Leibovitz's L15 (MDA-MB-231 and MDA-MB-453) or EMEM (BT20) medium supplemented with penicillin (100 IU/ml), streptomycin (100 µl/ml), L-glutamine (2 mM), and 10% heat inactivated fetal calf serum (Life Technologies), according to the supplier's recommendations. The monocytic cell line THP-1 was cultured in supplemented RPMI-1640 medium. Peripheral blood mononuclear cells (PBMC) were isolated from heparinized venous blood by density gradient centrifugation over LSM (EuroBio).

### Anti-CD160 antibodies

H3 monoclonal antibody (mAb; muIgG1) was generated by immunization of mice with a recombinant dimeric sCD160 protein and selected as described elsewhere [42]. H3 mAb was identified as recognizing both CD160-GPI and -TM isoforms. A12 antibody (huIgG1) is a fully humanized antibody obtained by phage display and selected for its specific reactivity with CD160-TM, but not CD160-GPI, -expressing cells by flow cytometry [42]. Epitope mapping revealed that A12 specificity relies on the recognition of a conformational epitope, with one moiety located within CD160-TM Ig domain and one mapping the extracellular membrane proximal region of the receptor (A12/epitopes 1 and 2; depicted in Additional file 1: Figure S2a). 22B12 mAb (muIgG2a) was generated by immunization of mice with specific peptides included within A12 epitope 2 (i.e. in the extracellular membrane-proximal region of CD160-TM). Its affinity for the immunizing peptides was verified by ELISA (Additional file 1: Figure S2b) while its specificity for CD160-TM was assessed by immunohistochemistry on transfected cells expressing either CD160-GPI or CD160-TM isoform (Additional file 1: Figure S2c). BY55 mAb and RD6700 are commercially available mAbs (R&D Systems) that allow detection of CD160-GPI, but not CD160-TM, by flow cytometry or immunohistochemistry, respectively ([42] and Additional file 1: Figure S2c).

### RNA extraction, reverse transcription and PCR

Total RNA was isolated using an extraction kit (RNeasy Plus Mini Kit, Qiagen) and reverse transcription was performed with the GoScript Reverse Transcriptase kit according to the manufacturer's recommendation (Promega). PCR was performed using Emerald PCR Master mix (Takara) and couples of primers allowing the specific detection of CD160-GPI or -TM full length and ∆Ig isoforms’ cDNA, as previously described [40]. The amplified products were separated on a 1% agarose gel and visualized with a FastGene Blue/Green LED GelPic Imaging System.

### Immunohistochemistry

Formalin-fixed paraffin-embedded skin biopsies or cell lines were labeled as previously described [42]. Briefly, 4 µM thick sections were subjected to antigen retrieval by heating in citrate buffer. Slides were then incubated with H3 or 22B12 mAb (1.1 µg/ml) followed by a secondary antibody and peroxidase, and detection with diaminobenzidine.

### Immunofluorescence

After adhesion on glass slides, TNBC cell lines were fixed in PBS/4% paraformaldehyde. After a blocking step in PBS/3% goat serum/3% BSA, anti-CD160 (H3 or 22B12 mAb) or isotype control antibodies (muIgG1 or muIgG2a) were added at a final concentration of 10 µg/ml. After washes, AlexaFluor 594- conjugated anti-mouse Igs antibodies (Invitrogen) were added. Slides were mounted with a DAPI Fluoromount solution (Southern Biotech).

### Flow cytometry

Cells were labeled with PE- or APC-conjugated anti-CD160 antibody (BY55 or A12) or their respective isotype-matched control antibodies. When indicated, cells were fixed and permeabilized prior to labeling according to the Cytofix/Cytoperm protocol (BD Biosciences).

After acquisition on a Cytoflex cytometer (Beckman Coulter), data analysis was performed using FlowJo software (Tree Star Inc.).

### Antibody-dependent cell phagocytosis (ADCP)

Target cells (TNBC cell lines) were labeled with CarboxyFluorescein Succinimidyl Ester (CFSE) according to the manufacturer's instruction (Invitrogen). THP-1 effector cells were then added at an effector to target (E/T) ratio of 1/1 together with 22B12 mAb (1 or 10 µg/ml) or Cetuximab (5 µg/ml) or the corresponding isotype-matched Ab (muIgG2a or huIgG1). After a 2.5 h incubation at 37 °C, cells were washed and labeled with an APC-coupled anti-CD33 mAb to delineate THP-1 cells. CFSE engulfment by CD33^+^ THP-1 cells was detected by flow cytometry and results expressed as the percentage of THP-1 CFSE^+^ cells among total CD33^+^ effector cells.

### Antibody-dependent cell cytotoxicity (ADCC)

TNBC cell lines expressing the green fluorescent protein (GFP) were generated by transfection using an eGFP Mission shRNA plasmid DNA (Sigma-Aldrich). Single spheroids were generated according to the manufacturer’s recommendations (Essen Bioscience/Sartorius) by plating cells in culture medium supplemented with 2.5% Matrigel (Corning). Spheroids formation and initial growth was monitored in a live imaging system (Incucyte; Essen Bioscience/Sartorius). After 4 days, PBMC were added together with IL2 (100 UI/ml) and IL15 (7.5 ng/ml) (Peprotech) plus the indicated antibody (10 µg/ml). Spheroids growth or shrinkage was monitored up to 8–10 days (one image every 6 h). Quantification of the fluorescence associated to each spheroid was performed and results expressed as the largest green surface area (mm^2^).

### TNBC mouse model

Eight-weeks old female SCID mice were engrafted intra-mammary with 5 × 10^6^ BT20 TNBC cell line. When tumors reached 65–100 mm^3^ (7 days post-engraftment), mice were separated in two groups (n = 5 per group). 22B12 mAb or isotype-matched negative control (muIgG2a) was administrated intra-peritoneally twice a week for 2 weeks (at day 7, 10, 14 and 17). Tumor size was measured prior to Ab inoculation and tumor volume (in mm^3^) was calculated as (length × width^2^)/2.

#### Statistical analysis

For each experiment, results are expressed as means ± SD of 3 to 5 independent experiments or triplicates. Statistical analyses were performed using a Mann–Whitney *t* test with Prism GraphPad software. *p* < 0.05 was considered as significant.

## Results

### Expression of CD160 by TNBC

**Fig. 1:**
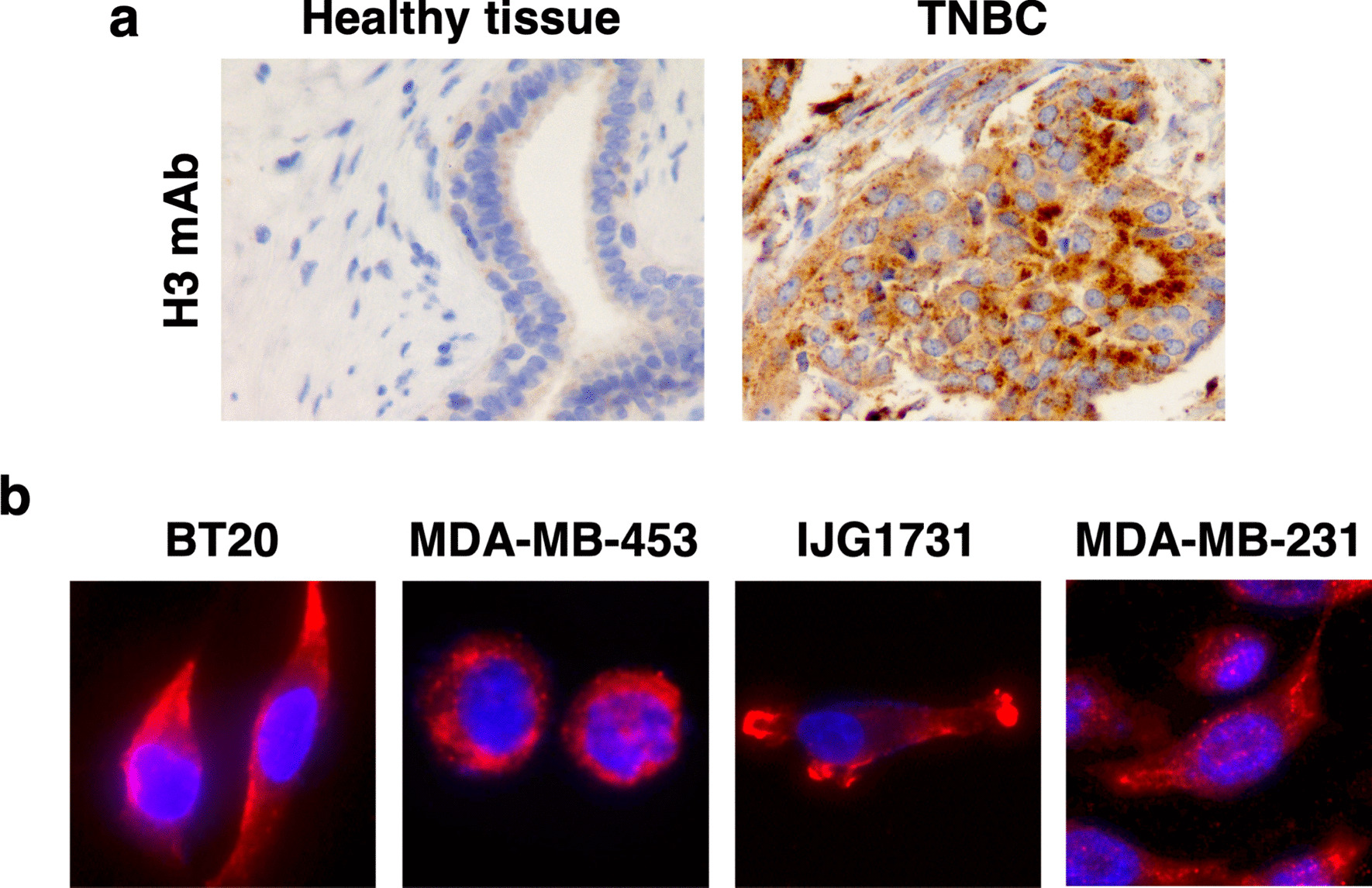
Expression of CD160 by TNBC tumor cells. **a** Healthy (n = 3) or tumor (n = 3) tissue from TNBC patients was subjected to immunostaining using anti-CD160 mAb H3. Displayed images are from representative samples. **b** After adhesion on glass slides and fixation, TNBC cell lines were incubated with anti-CD160 mAb H3 followed by AlexaFluor594-coupled anti-mouse Igs antibodies. Slides were mounted with a Dapi-containing medium for nuclei counterstaining (blue). Images were acquired on a Nikon Ni fluorescence microscope. Magnification: 60×

Our previous studies established that, while CD160-GPI is constitutively expressed by NK cells, CD160-TM expression is induced upon NK cell activation [19, 40]. We therefore thought that detection of one or both isoforms might be a reliable marker for evaluation of NK cells’ infiltration within solid tumors. We analyzed several types of tumor biopsies by immunohistochemistry using anti-CD160 H3 mAb, previously characterized as able to recognize both CD160-GPI and CD160-TM isoforms [42]. A strong and positive signal was detected in biopsies from TNBC patients but not in healthy breast tissues. However, this signal appeared not to delineate immune cell infiltrates but rather the tumor cells (Fig. 1a). This assumption was further investigated by performing immunofluorescence labeling on TNBC cell lines using H3 mAb. As shown in Fig. 1b, a positive signal was detected with a cytoplasmic and/or plasma membrane location in all TNBC cell lines tested (Fig. 1b), confirming that CD160 could be constitutively expressed by TNBC tumor cells.

### TNBC cell lines only express CD160-TM isoform

**Fig. 2:**
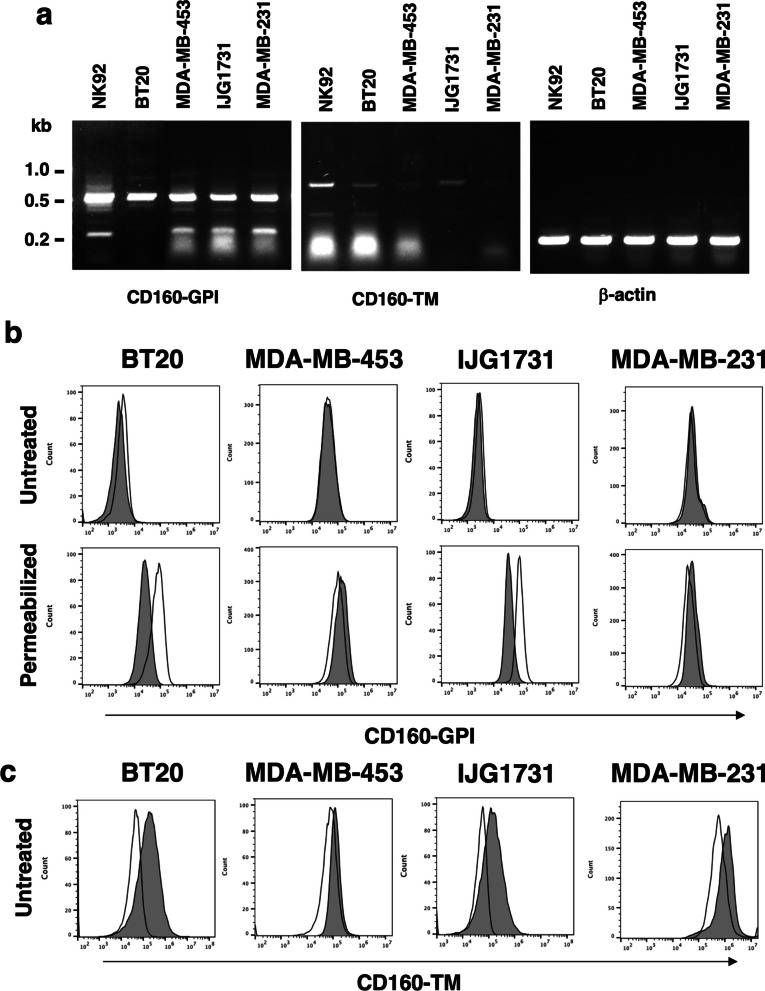
Expression of CD160-TM isoform by TNBC cell lines. **a** Total RNAs were extracted from TNBC (BT20, MDA-MB-453, IJG1731 and MDA-MB-453) and NK92 cell lines and subjected to reverse transcription. PCR were realized using a pair of primers corresponding to the 5′ and 3′ ends of CD160-GPI or CD160-TM reported coding sequence. Amplification of β-actin cDNA was performed in parallel as an internal control. **b** Analysis of CD160-GPI expression by TNBC cells. Cells were either left untreated (top panels) or subjected to a fixation/permeabilization step prior to labeling (bottom panels). Staining was performed using a PE-coupled anti-CD160-GPI mAb (BY55; grey histograms) or isotype control Igs (muIgM; white histograms). Cells were then analyzed by flow cytometry. **c** Analysis of CD160-TM expression by TNBC cells. Cells were labeled using APC-coupled anti-CD160-TM Ab (A12; grey histograms) or the corresponding isotype control (huIgG1; white histograms) and analyzed by flow cytometry

Because H3 mAb could not discriminate between the GPI and TM isoforms of CD160, we underwent a more precise analysis of CD160 expression in TNBC cell lines. RT-PCR was first performed on total RNA extracted from TNBC cell lines (BT20, MDA-MB-231, IJG1731 and MDA-MB-453) for detection of CD160-GPI and CD160-TM transcripts. Total RNA from the NK cell line NK92, previously shown to constitutively express CD160-GPI and -TM transcripts, was used as positive control [40]. Two transcripts of 550 and 220 bp, corresponding to the full length and ∆Ig (devoid of Ig domain coding sequence) isoform cDNA of CD160-GPI respectively, were found in all TNBC cell lines tested. Similarly, full length (700 bp) or ∆Ig (380 bp) CD160-TM encoding transcripts were detected (Fig. 2a).

To determine if the synthesis of CD160 transcripts by TNBC cells was correlated with efficient protein expression, flow cytometry analysis was undertaken using antibodies specific for CD160-GPI (BY55 mAb) or CD160-TM (A12 Ab) isoform [42]. As shown in Fig. 2b, no expression of CD160- GPI was detected at the cell surface of TNBC cell lines (Fig. 2b, upper panels). In addition, no CD160-GPI-associated signal was observed at the intracellular level following cell permeabilization (Fig. 2b, lower panels). In contrast, a positive but heterogenous signal was observed on live cells when using A12 anti-CD160-TM Ab (Fig. 2c). Indeed, while CD160-TM was detected at the plasma membrane of BT20 and IJG1731, and to a lower extent MDA-MB-231 cells, it was undetectable on MDA-MB-453 cell line. Regarding this latter cell line, one cannot exclude a mainly cytoplasmic expression of CD160-TM, a possibility supported by the results of the above IF labeling where CD160-associated signal appeared more cytoplasmic than plasma-membrane associated in this cell line (see Fig. 2b). Nevertheless, this set of data ruled out an expression of CD160-GPI isoform and favored an exclusive expression of CD160-TM isoform by TNBC tumor cells.

### Generation and characterization of novel anti-CD160-TM mAb with a single epitope-based recognition site

Although A12 Ab was characterized as a specific anti-CD160-TM Ab [42], allowing detection of the receptor on a subset of activated NK and CD8^+^ T lymphocytes (Additional file 1: Figure S1), epitope mapping revealed that its specificity relies on the recognition of a conformational epitope (depicted in Additional file 1: Figure S2a), therefore leading to some experimental limitations. Indeed, it became ineffective for CD160-TM detection in non-native conditions (e.g. after cell fixation, permeabilization or paraffin inclusion; data not shown), preventing the easy and unequivocal demonstration of CD160-TM expression by IHC on TNBC patients' tumor biopsies. An immunization program was therefore conducted for obtaining novel monoclonal Abs directed against a linear and specific epitope of CD160-TM. Mice were immunized with peptides located within the membrane-proximal region of the TM isoform (within the previously identified A12 epitope 2) and corresponding to an amino-acids stretch that is cleaved at the time of the GPI adjunction process in CD160-GPI (see Additional file 1: Figure S2a).

The obtained mAbs were then tested for their ability to: (i) recognize the immunizing linear peptides, (ii) specifically detect CD160-TM on paraffin-embedded cells (to further ensure their use on patients' tumor biopsies) and (iii) trigger CD160-TM functional activity on NK cells and therefore induce an amplification of NK cell cytotoxic activity [40]. One antibody, called 22B12, fulfilled these three criterions. Indeed, it strongly recognized the immunization peptides (Additional file 1: Figure S2b) and allowed detection of CD160-TM, but not CD160-GPI, on paraffin-embedded cells forced to express one or the other isoform (Additional file 1: Figure S2c). 22B12 also showed an agonist activity as it enhanced NK cell killing activity toward the HLA-negative target cells K562 (Additional file 1: Figure S2d) and amplified the ADCC process mediated by the anti-CD20 Ab Rituximab against Raji B cells (Additional file 1: Figure S2e).

**Fig. 3:**
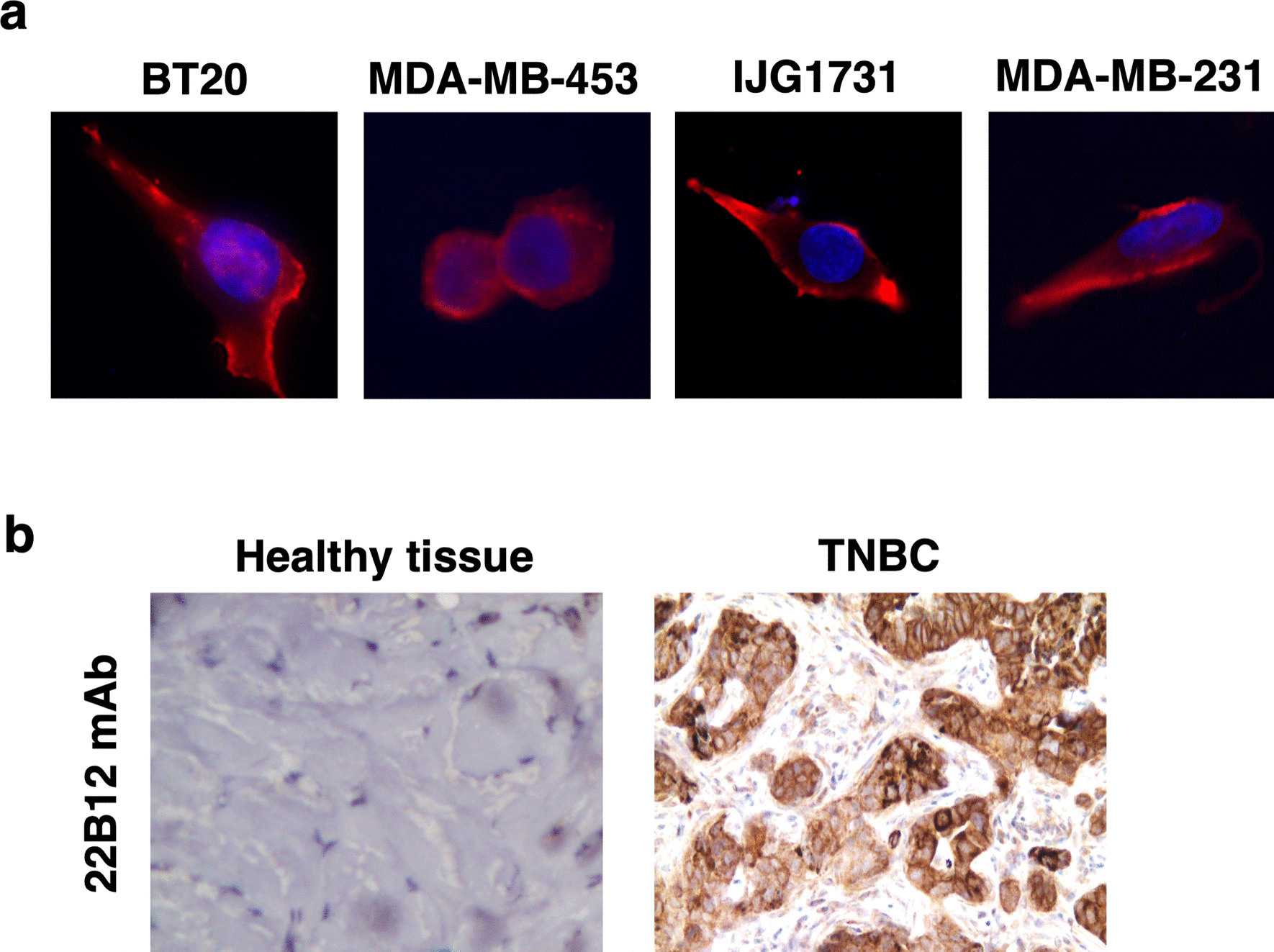
Validation of 22B12 mAb for detection of CD160-TM in TNBC tumor cells. **a** After adhesion on glass slides and fixation, TNBC cell lines were incubated with anti-CD160 mAb 22B12 followed by AlexaFluor594-coupled anti-mouse Igs antibodies. Control labeling with muIgG2a Ig was performed in parallel. Slides were mounted with a Dapi-containing medium for nuclei counterstaining (blue). Images were acquired on a Nikon Ni fluorescence microscope. Magnification: 60×. **b** Healthy (n = 4) or tumor (n = 10) tissue from TNBC patients was subjected to single immunostaining using 22B12 mAb. Shown are images from a representative sample (full set of images available in Additional file 1: Figure S3)

22B12 specificity for CD160-TM being established, we next tested its ability to detect CD160-TM when expressed by TNBC. Immunofluorescence labeling was performed on TNBC cell lines and IHC experiment was conducted on TNBC patients’ biopsies (n = 10; Fig. 3 and Additional file 1: Figure S3). In both settings a positive signal was detected with 22B12 mAb, resembling the one previously obtained with H3 mAb (Fig. 2).

Altogether, these results validate 22B12 mAb as allowing in vitro and in situ specific detection of CD160-TM in TNBC cell lines and tumor biopsies respectively.

### 22B12 anti-CD160-TM mAb mediates ADCP or ADCC toward TNBC cell lines in vitro

**Fig. 4:**
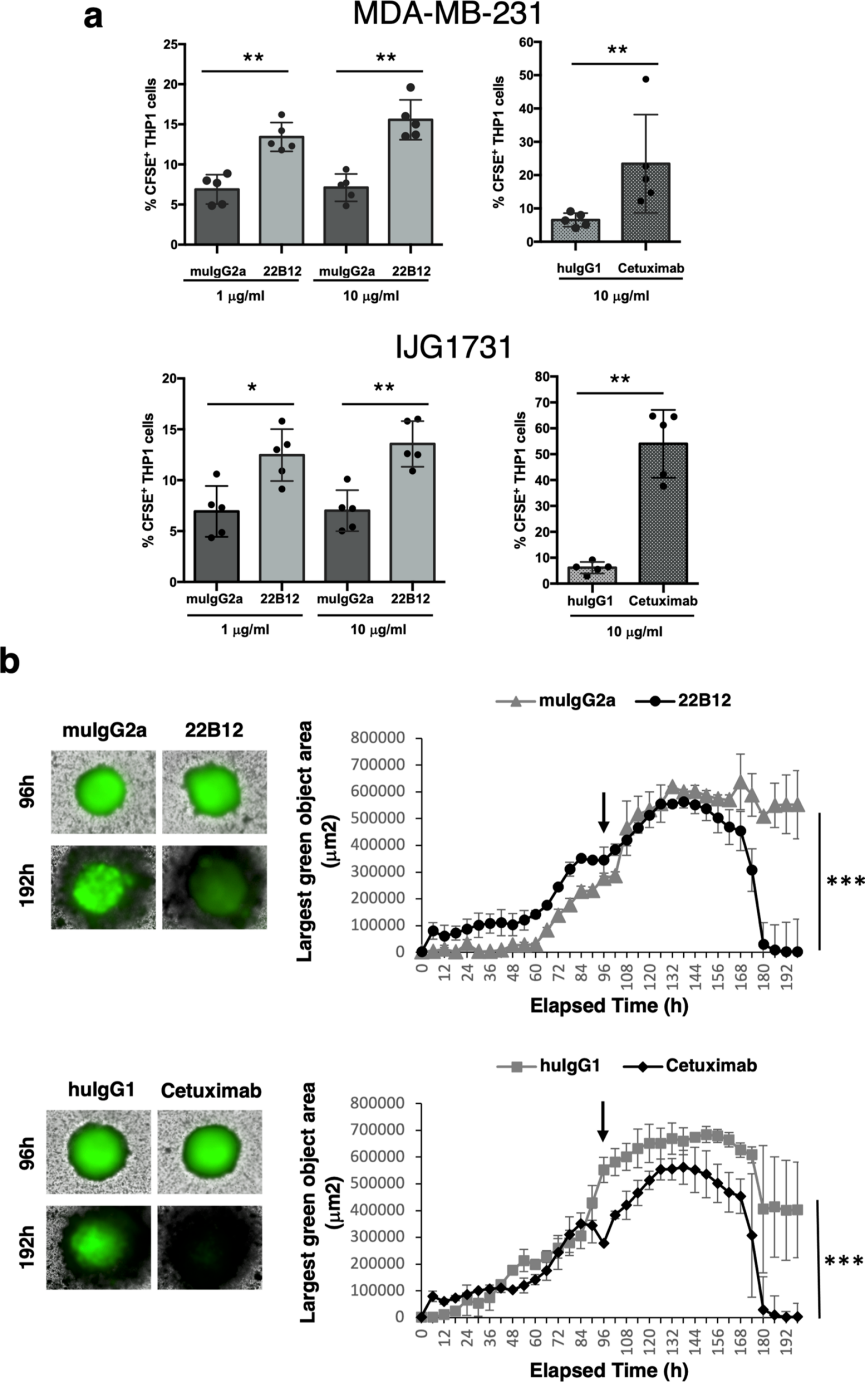
Promotion of ADCP and ADCC by 22B12 mAb. **a** CFSE-labeled TNBC cell line (MDA-MB231 or IJG1731) was mixed together with THP-1 at an E/T ratio of 1/1 together with isotype control or 22B12 mAb. The anti-EGFR Ab Cetuximab (huIgG1 isotype) was used as positive control. After 2.5 h of cell contact, phagocytosis was evaluated by flow cytometry by detecting the % of THP-1 cells that became CFSE^+^. Results are expressed as the mean ± SD of 3 independent experiments. *p < 0.05; **p < 0.01. **b** Single spheroids were obtained from IJG1731 cells stably transfected with GFP. After 4 days of growth (arrow), PBMC were added together with a mix of IL2 + IL15 and 22B12 mAb, Cetuximab, or their corresponding isotype-matched Abs. The green fluorescence associated to each spheroid was monitored every 6 h for 4 additional days in a live cell imaging system. *Left panels:* images of a representative spheroid immediately after addition of PBMC and the indicated Abs (96 h) and at the end of the assay (192 h). *Right panels:* quantification of the green fluorescence associated to each spheroid over time. Results are expressed as the mean ± SD of triplicates. ***p < 0.001

The identification of CD160-TM as a potential tumor target on TNBC cells prompted use to investigate the ability of 22B12 to mediate TNBC tumor cell depletion. The promotion of antibody-dependent cell phagocytosis (ADCP) was first evaluated by using the monocytic cell line THP-1 as effector cells and TNBC cell lines as target cells. MDA-MB-231 and IJG1731 were selected to assess tumor depletion on low and high CD160-TM expressing cells, respectively (see expression level in Fig. 2c, corresponding to A12/control Ab MFI ratio = 2.2 and 8.9 for MDA-MB-231 and IJG1731, respectively). The anti-EGFR antibody Cetuximab was used as positive control as EGFR expression was detected on both target cells by prior phenotyping. As shown in Fig. 4a, phagocytosis of TNBC cells was induced in the presence of 22B12 mAb. Thus, a statistically significant engulfment of TNBC cells by THP-1 cells was detected, with maximum levels reached at 1 or 10 µg/ml depending on the cell line.

Beside ADCP, NK cell-mediated tumor cell depletion by antibody-dependent cell cytotoxicity (ADCC) was also tested in a three-dimensional functional assay. To this end, a GFP-expressing IJG1731 cell line was established for further use as target cells. Spheroids were generated from GFP^+^ IJG1731 cells and co-cultured with PBMC isolated from the blood of healthy volunteers (as source of effector cells) in the presence of IL15 and IL2. Following addition of the antibodies, spheroids integrity was assessed by using a live imaging system set to monitor the green fluorescence associated to each spheroid every 6 h. As demonstrated in Fig. 4b, both 22B12 (upper panel) and Cetuximab (lower panel) induce an immune-dependent loss of viability of the spheroids. Indeed, a total loss of the spheroid-associated green fluorescence was observed with both Abs when compared to their respective isotype control (*p* < 0.001).

### In vivo validation of 22B12 mAb anti-tumor efficacy in a TNBC murine model

**Fig. 5:**
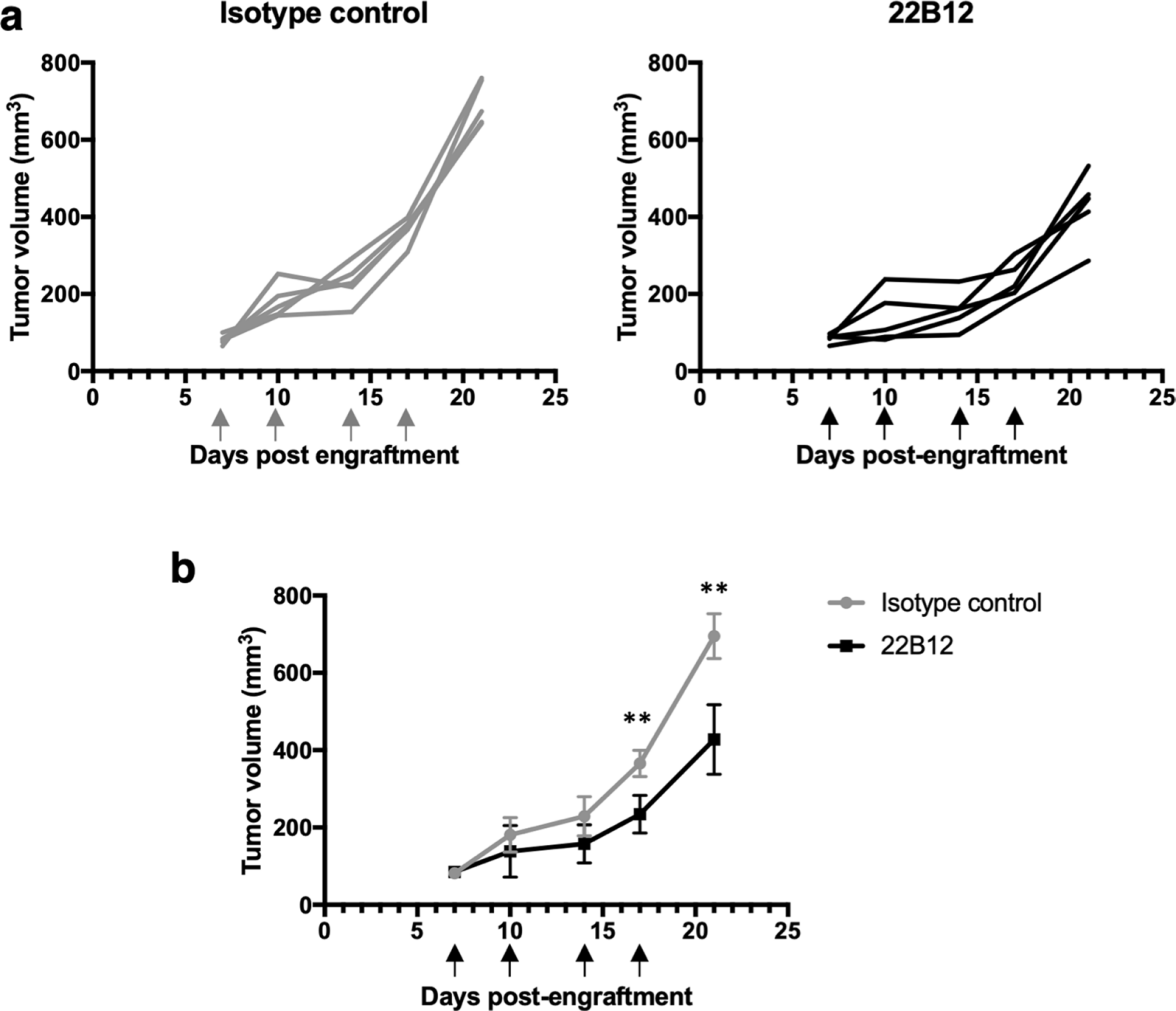
In vivo anti-tumor efficacy of 22B12 mAb. Mice were engrafted intra-mammary with BT20 cell line. After tumor growth, five mice per group received either 22B12 mAb or its corresponding isotype control (100 µg per injection twice weekly for two weeks). **a** Individual tumor growth in mice from the control (left) or 22B12 (right) group. **b** Same data as in (**a**) but expressed as the mean ± SD of each group over time. Arrows: mAb injections. Statistical comparison was performed with a Mann–Whitney *t* test. **p = 0.008 when compared to the control group

To study the potential anti-tumor function of 22B12 mAb in vivo, we used a xenograft mouse model of CD160-TM^+^ TNBC tumor in the SCID mouse, known to retain partially functional NK cells and fully functional macrophages [43]. A therapeutic setting has been designed, with injection of the Ab once the tumors have been established, in order to better reflect the tumor reality encountered by patients. To this end, BT20 TNBC cells were injected intra-mammary and 22B12 mAb or its corresponding isotype control (muIgG2a) was administered intra-peritoneally twice weekly for 2 weeks (100 µg per injection), starting when tumors reached 65–100 mm^3^ (7-days post-engraftment). Figure 5a shows the individual tumor growth curves in both treatment groups, with a significantly reduced tumor growth in the 22B12-treated group when compared to the control group. Notably, all animals from the control group presented significantly larger tumors than the ones from the 22B12 group with a significant growth reduction being detected after the third injection of anti-CD160-TM mAb (17-days post-engraftment; *p* < 0.01) that persisted thereafter (Fig. 5b). This in vivo experiment therefore demonstrated that 22B12 mAb exerts anti-tumor efficacy in a TNBC tumor model.

## Discussion

Over several decades, antibody-based therapies have revolutionized the management and prognosis of many cancers. Among the currently used therapeutic antibodies that promote tumor cell targeting and depletion, one can cite the anti-CD20 Rituximab for B cell lymphoma [44], the anti-EGFR Cetuximab for colorectal, head and neck or lung cancers [45, 46], and the anti-KIR3DL2 Lacutamab or anti-CCR4 Mogamulizumab for cutaneous T cell lymphoma [47, 48]. Although resistance or relapse could be observed upon such treatment, the identification of tumor-membrane antigen and the generation of anti-tumor antigen antibodies remained a major challenge for the development of novel therapeutic options, especially in cancers for which no tumor specific marker was identified so far. Regarding breast cancers, while the survival outcome of hormone receptor- or HER2-positive BC has been improved by the development of HER2-targeting mAbs or antibody–drug conjugates, the lack of targetable cell surface antigen has deeply hampered the development of anti-tumor antibody-based therapies for TNBC. We here reported the identification of CD160-TM as a tumor marker for TNBC as well as the generation of a specific anti-CD160-TM mAb able to promote TNBC cell depletion.

The nearly complete identity of the extracellular domains of CD160-GPI and -TM isoforms has long represented an obstacle for the obtention of Abs allowing the unequivocal recognition of each isoform. Thus, currently available Abs (generated in-house or commercially available) either recognized both molecules (e.g. H3 mAb) or the GPI form only (as BY55, RD6700 or CL1-R2 mAb) [19, 20, 22, 42]. We recently reported the first Ab recognizing CD160-TM, but not CD160-GPI, namely A12 Ab [42]. A12 recognition site was identified as encompassing two amino-acid stretches located on the N- and C-terminal sides of CD160-TM extracellular domain. Consequently, its binding to the TM receptor was found strictly conformation-dependent. If ensuring its specificity for detection of CD160-TM expression on live cells by flow cytometry (Fig. 2 and Additional file 1: Figure S1), this characteristic rendered A12 Ab inadequate for assays where total or partial protein denaturation was required (e.g. IHC or IF). The use of linear peptides located within A12 C-terminal epitope, and therefore within the membrane-proximal sequence unique to CD160-TM, allowed the selection of the novel specific CD160-TM mAb termed 22B12. As expected, 22B12 mAb allowed CD160-TM detection on paraffin-embedded or fixed cells, and more importantly on TNBC patients' tumor biopsies but not on healthy breast tissues, suggesting that it might represent a valuable tool for TNBC pre-therapeutic phenotyping. Beside its specificity, we also established that 22B12 mAb presented efficient anti-tumor activity as it promoted NK cell- or phagocyte-mediated TNBC cells depletion in vitro. Additionally, its injection in a TNBC mouse model, using a therapeutic setting, resulted in a significant reduction of the tumor growth, supporting the idea that 22B12 promoted the establishment of an anti-tumor immune response. Although our data provided a rational for developing an anti-CD160-TM antibody-based therapy for TNBC, several steps will remain necessary to definitely validate such approach. Generation of a chimeric anti-CD160-TM, combining the variable domains of 22B12 to the constant domain of human IgG1 is currently in progress. The resulting Ab (22B12-huIgG1) would then have to be tested for its ability to promote in vitro ADCP and ADCC and in vivo control of tumor growth, as described herein. In addition, its evaluation in combination with other chemo-therapeutic agents or immuno-therapies will also be considered.

Immune cells phenotyping performed on PBMC with A12 Ab confirmed CD160-TM expression on activated NK but also on a subset of CD8^+^ T cells, while CD4^+^ T cells were identified as CD160-TM negative cells (Additional file 1: Figure S1). We demonstrated that addition of 22B12 mAb to NK cells led to an enhancement of their natural cytotoxicity towards target cells (Additional file 1: Figure S2d). One can therefore anticipate that 22B12 mAb could exert a double-trigger effect by, on one hand, promoting TNBC tumor cell targeting and, on the other hand, boosting NK cell (and potentially CD8^+^ T cells) cytotoxicity. Although validation of this hypothesis will need further investigations, it is currently supported by our preliminary data showing in an ADCC functional assay that depletion of the CD20^+^ B cell line Raji by Rituximab was amplified in the presence of 22B12 mAb (Additional file 1: Figure S2e).

In conclusion, our data described the identification of the first tumor antigen for TNBC. The obtention of a specific CD160-TM, able to promote targeted tumor cell depletion, opens new and attractive perspectives for the development of anti-CD160-TM therapeutic antibodies in the current field of TNBC treatments.

### Supplementary Information

**Additional file 1.** Supplementary Figures 1–3.

## Data Availability

Data are available upon reasonable request.

## Abbreviations

BC: Breast cancer
TNBC: Triple-negative brest cancer
GPI: Glycosyl-phosphatidyl-inositol
TM: Transmembrane
ADCC: Antibody-dependent cell cytotoxicity
ADCP: Antibody-dependent cell phagocytosis
Ig: Immunoglobulin
Ab: Antibody
HR: Hormone receptor
PR: Progesterone receptor
ER: Estrogen receptor
HER2: Human epidermal growth factor receptor-2
EGFR: Epidermal growth factor receptor
IL: Interleukin
HVEM: Herpes virus entry mediator
PBMC: Peripheral blood mononuclear cells
IHC: Immunohistochemistry
IF: Immunofluorescence
