# Supplementary material for: Identification of CD160-TM as a tumor target on triple negative breast cancers: possible therapeutic applications
Source: Breast Cancer Res. 2024 Feb 15;26:28. doi: 10.1186/s13058-024-01785-x (PMC10870674; doi:10.1186/s13058-024-01785-x)

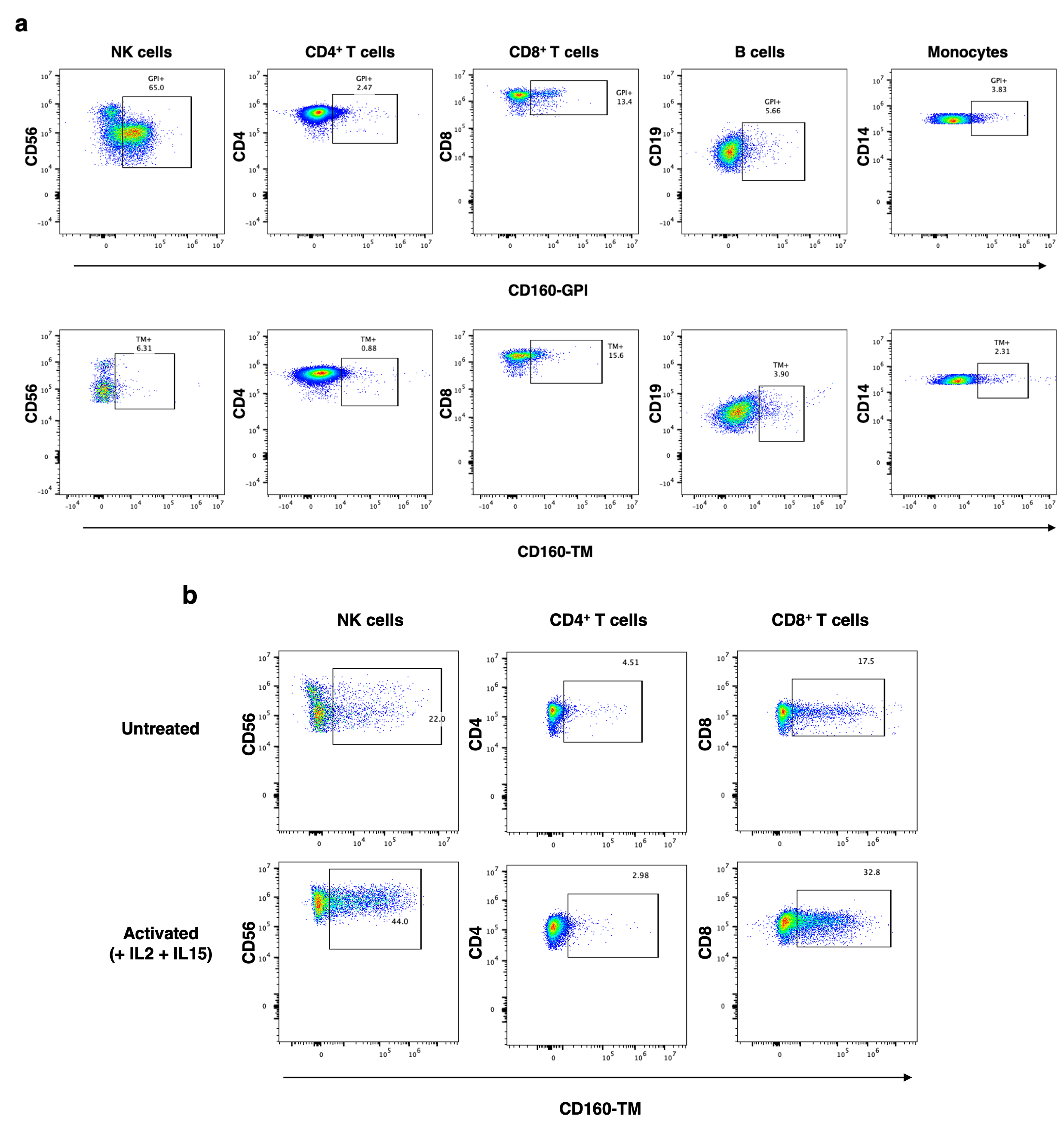


**Supplemental Figure S1: Expression profile of CD160-GPI and CD160-TM on immune cells. (a) CD160 isoforms expression on resting cells.** Freshly isolated PBMC were subjected to 9-color immuno-labeling using a mix of fluorochrome-conjugated anti-CD3, -CD4, -CD8, -CD14, -CD19, -CD45, -CD56, CD160-GPI (BY55) and CD160-TM (A12) antibodies. Respective isotype-matched antibodies were used as negative controls for background thresholds determination (fixed at < 6% for both GPI and TM labeling). Cells were analyzed by flow cytometry. Following FSC/SSC (lymphocytes *vs* monocytes) and CD45^+^ gating, expression of CD160-GPI (upper panels) and CD160-TM (lower panels) was determined on the NK (CD3^-^CD56^+^), T (CD3^+^CD4^+^ or CD3^+^CD8^+^), B (CD3^-^CD19^+^) and monocytes (CD14^+^) populations. Shown are results corresponding to a representative blood donor. **(b)** **Induction of CD160-TM expression upon activation**. PBMC were either left untreated (upper panel) or incubated with a mixture of IL2 (100UI/ml) and IL15 (7.5 ng/ml) (lower panels) for 4 days. Cells were then labeled with fluorochrome-conjugated anti-CD3, -CD4, -CD8, -CD45 and -CD160-TM (A12) antibodies. Controls and subsequent analyses were performed as described in **(a)**.


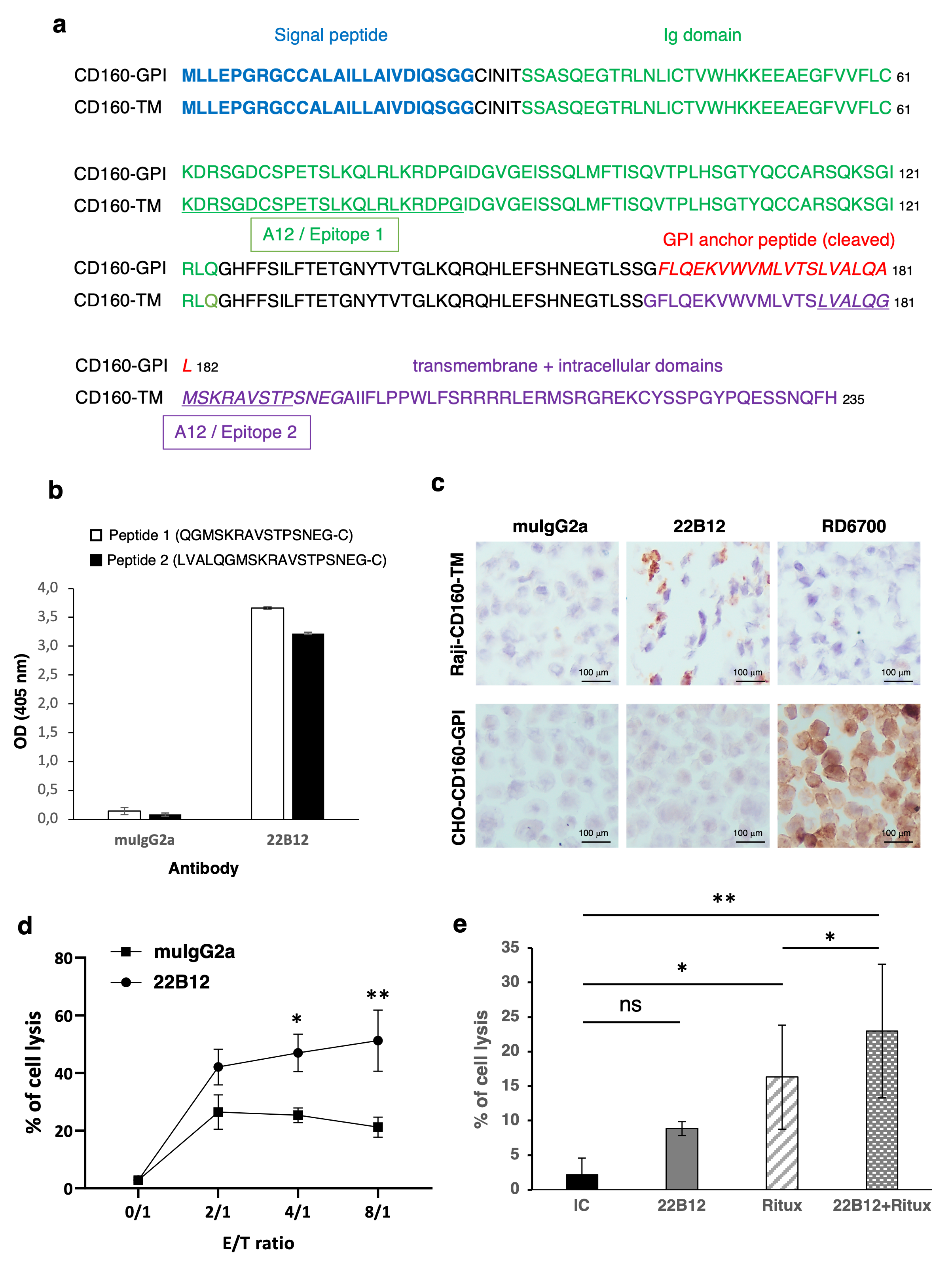


**Supplemental Figure S2: Generation and selection of novel anti-CD160-TM mAb.** **(a)** Amino-acids sequences of CD160-GPI and CD160-TM isoforms. The recognition sequences of A12 Ab, identified by epitope mapping and located within CD160-TM Ig and juxta-membrane domains (epitope 1 and 2, respectively), are underlined. The amino-acid sequence cleaved upon the addition process of the GPI anchor is highlighted in red. **(b)** Recognition of the peptides used for mice immunization by 22B12 mAb. Isotype control mAb (muIgG2a) was used as negative control. **(c)** Immunolabeling of CD160-GPI or CD160-TM paraffin-embedded cells with 22B12 mAb. Raji or CHO cells were transfected with a vector coding for the TM or GPI isoform, respectively. Cell pellets were embedded in paraffin and labeling was performed on 4 μM sections using a control isotype (left panels), 22B12 mAb (middle panels) or a commercially available anti-CD160 mAb (RD6700). **(d)** Amplification of NK cell natural cytotoxicity by 22B12 mAb. Cytotoxicity assays were performed using NK92 cell line (constitutively expressing CD160-TM) as effector (E) cells and the HLA-negative cell line K562 as target (T) cells. Cells were mixed together at the indicated E/T ratio in the presence of muIgG2a isotype control or 22B12 mAb. After 4 h of incubation, cells were labeled with 7AAD and analyzed by flow cytometry for detection of 7AAD^+^ cells among K562 cells**. (E)** Amplification of Rituximab-mediated ADCC by 22B12 mAb. PBMC and Raji cells were mixed at an E/T ratio of 20/1 together with 22B12 and Rituximab alone or in combination. Detection of target cell apoptosis was performed as in (D). (D and E) Shown are results corresponding to the mean ± SD of 3 independent experiments. Statistical analysis was made using a Mann-Whitney test. * p<0.05, ** p<0.01.


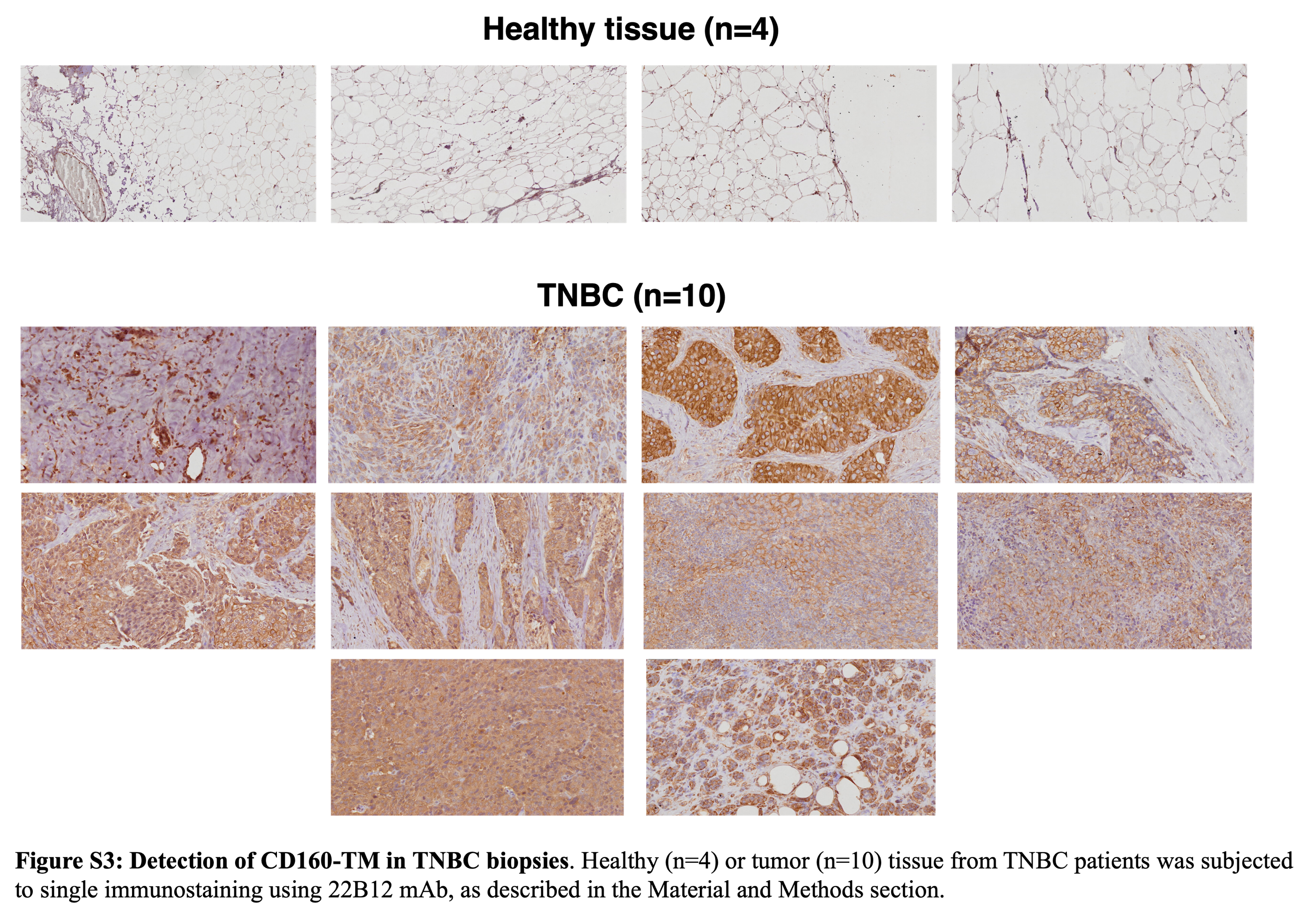

Supplement: Supplementary file 1 — Additional file 1. Supplementary Figures 1–3. [file 13058_2024_1785_MOESM1_ESM.docx]
